# Sociodemographic and clinical characteristics and access to health care in patients with spinal muscular atrophy in Argentina

**DOI:** 10.3389/fneur.2023.1179692

**Published:** 2023-09-07

**Authors:** Gabriel Adolfo Vazquez, Salomé Nasif, Sebastián Marciano, Vanina Pagotto

**Affiliations:** ^1^Department of Pediatric Neurology, Sanatorio Güemes, Buenos Aires, Argentina; ^2^Neuropediatrics Program, Sanatorio Güemes - Universidad de Buenos Aires, Buenos Aires, Argentina; ^3^Department of Neuropathology and Genetics, Universidad Nacional de Tres de Febrero, Buenos Aires, Argentina; ^4^Argentine Society of Child Neurology (SANI), Buenos Aires, Argentina; ^5^Hospital de Niños Dr. Pedro de Elizalde, Buenos Aires, Argentina; ^6^International Parkinson and Movement Disorders Society (MDS), Buenos Aires, Argentina; ^7^Research Department of the Hospital Italiano de Buenos Aires, Buenos Aires, Argentina

**Keywords:** spinal muscular atrophies, hipotony, SMA, standar of care, FAME

## Abstract

The FAME registry gathers the majority of patients with SMA in Argentina. From it, the clinical, sociodemographic and access to treatment characteristics were analyzed in 322 patients (range 8 months-61 years) included from 2008 to 2021. Important data were obtained for the planning of medical care of these patients such as: similar distribution of patient care in public and private hospitals, time gap between onset of symptoms and diagnoses, low level of completion of *SMN2* copy count, estimate of 16 new diagnoses per year between 2014 and 2018, and 68% of patient in specific pharmacological treatment.

## 1. Introduction

Spinal muscular atrophy 5q- (SMA) is an autosomal recessive disease mapped to the long arm of chromosome 5 (1). In recent years, dramatic progress has been made in both specific treatment and standards of care (2–4). One of the pillars of the community approach to the disease is knowledge of its own characteristics in different countries and regions (5, 6).

In the case of Argentina, there is currently no centralized patient registry, so they are in charge of patient groups or some health institutions. On the other hand, there is an organized patient association with a presence in different medical, social and academic fields (Familias AME Argentina - FAME -) that has its own patient registry. This association nucleates the vast majority of patients with the disease. The association has had 350 registered patients since 2008. Until the present work, the registry information had not been systematically analyzed within the framework of a research protocol (7).

There is no centralized registry of the Ministry of Health for which the analysis carried out is of fundamental importance for health care planning, treatment access and newborn screening.

In Argentina, the three specific drugs for the disease were approved by the regulatory entity (ANMAT: national administration of medicines, food and medical technology): Nusinersen (2019), Onasemnogene (2021) and Risdiplam (2022). In the present work, it was analyzed which group of patients receives them, leaving for a second stage specific studies of accessibility, specific indications or therapeutic results with them (8–10).

Although Argentina has a long tradition of neonatal screening for various pathologies, SMA is not included in it (11). Despite this, our group has participated in the global consortium on neonatal research since its creation (12).

## 2. Materials and methods

1) Patients are voluntarily included in the registry from annual meetings of the association, its web page, telephone contact with them or their families. The data is obtained from a semi-structured questionnaire self-administered by patients or families, without medical intervention in the process. There was no rejection of them when invited to enter the registry.
2) This study is cross-sectional, analyzing specific data at the end of it. In case of need to corroborate or update data, telephone or email contact was established. The filiation data were coded to guarantee confidentiality.
3) The data were analyzed by a specialist in neuromuscular diseases.
4) Inclusion criteria: availability of confirmatory genetic test for SMA 5q.
5) Exclusion criteria: incomplete or inconsistent data from the medical point of view after analysis by a specialist.
6) Demographic variables: age, sex, place of residence and medical care, type of health coverage (classified as public, prepaid medicine and social security, according to the characteristics of the Argentine health system), eventual date and age of death.
7) Clinical variables: SMA subtype, age and diagnostic delay, *SMN2* copy count, greatest motor capacity achieved and greatest current motor capacity (according to the WHO motor milestone scale), need and type of respiratory support, need for nutritional support.
8) Variables of access to treatment: access to physical therapy, cough assistance, and other therapies.
9) Mortality curve is analyzed and incidence and prevalence are estimated, using data from INDEC (National Institute of Statistics and Censuses).
10) Descriptive statistics were used, with absolute numbers and percentages for categorical variables, and median and interquartile ranges (or percentiles) 25–75% for numerical variables. Statistical analysis was performed with R software version 4.0.

## 3. Results

### 3.1. Patients included and excluded sociodemographic data

In this work 332 patients were included, out of a total of 350 records analyzed. Those excluded (18%) were mainly due to the lack of a confirmatory molecular study, medically inconsistent data (report of SMA2 with independent gait) or impossibility of corroborating them (deceased patient and family with whom contact cannot be established). Of the 332 patients, 172 are male (51.8%).

Regarding the SMA subtype: 99 (29.8%) are SMA 1, 149 (44.9%) are SMA 2, 81 (24.4%) are SMA 3 and 3 (0.9%) are SMA 4. The current median age of the patients and the interquartile ranges (IQR) expressed in years are for type 1 SMA 3.1 (3.1–6.6), for type 2 SMA 14.2 (8.18–22.9), for type 3 SMA 19.2 (12.9–32.7) and for type 4 SMA 58.2 (57.08–60.5).

Globally, 44% of patients are younger than 10 years, 27% are between 10–19 years, and 28% are 20 years or older. The age ranges in SMA 1 are 1.8 months to 20 months, with 11 patients older than 10 years, which makes a percentage of 11.1% in this age range (10 patients between 10 and 19 years and one 20 years old).

In the case of SMA 2, the range is from 1 year and 2 months up to 67 years. There are 51 patients with more than 20 years (34.2%), between 10 and 19 years 52 patients (34.8%) and between 1 and 9 years there are 46 patients (30.8%) (Table 1).

**Table 1:** Characteristics of the included patients.

	SMA 1	SMA 2	SMA 3	SMA 4
Number	99 (29.8%)	149 (44.9%)	81 (24.4%)	3 (0.9%)
Gender	F 43 (43.4%) M 56 (56.6%)	F 69 (46.3%) M 80 (53.7%)	F 47 (58.0%) M 34 (42.0%)	F 1 (33.3%) M 2 (66.7%)
Disease	32	1	1	0
Deceased in first year of life	17	0	0	0
Current age in years, median age and IQR	3.1 m (3.10–6.6)	14.20 m (8.18–22.94)	19.2 m (12.9–32.7)	58.27 years (57.08–60.58)
Age of onset in months - years, median age and IQR	3.00 m (2.00–4.00)	11.7 m (7.00–15.00)	24.00 m (8.00–51.00)	18 years (18–19)
Age of diagnosis in months - years, median age and IQR	5.2 m (3.37–7.43)	18.68 m (14.18–27.77)	49.87 m (29–118)	28 years (26–34)
Age range	8 m-20 months	1–67 months	3–61 months	56–63 years
Patients under 10 years	88	46	13	0
Patients between 10 and 19 years	10	52	30	0
Patients over 20 years	1	51	38	3
copies of SMN2 (only 33.4% of the total)	19 two copies	16 two copies 32 three copies	17 three copies	UNK

SMA, spinal muscular atrophy; F, female; M, male; IQR, interquartile range; m, months; UNK, unknown.

The range of patients with SMA 3 is from 3 years to 61 years, 13 patients (16%) are younger than 10 years, 30 patients are between 10 and 19 years (37%) and 38 patients (47%) are 20 years or older.

**Figure 1:**
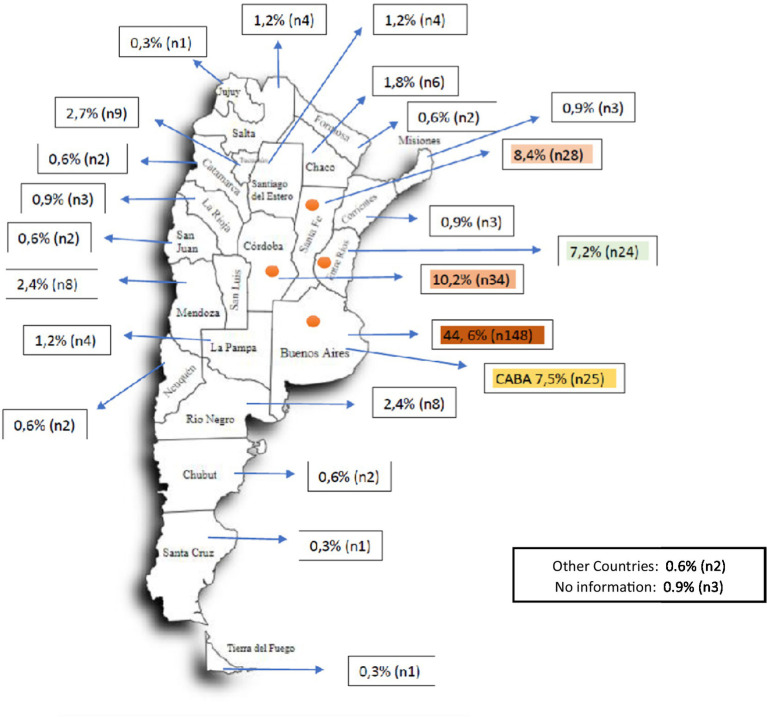
Geographical distribution of patients by place of residence. CABA: Autonomous City of Buenos Aires. The colored provinces (CABA, Buenos Aires, Entre Ríos, Córdoba, Entre Ríos) represent the districts with the highest number of patients with SMA. Together with Mendoza, they represent the states that accumulate 69.77% of the country’s inhabitants (27,993,571 out of a total of 40,117,096 inhabitants).

Of the 332 patients, 175 correspond to the area of the Province of Buenos Aires (PBA) and the City of Buenos Aires (CABA), constituting 52% of the cases. If the provinces of Córdoba, Santa Fe and Entre Ríos are added, this entire group of provinces concentrates 78% of the country’s cases (Figure 1).

In relation to the care system 50.6% of patients are cared for in the social security system, 24.1% in prepaid medicine and 22% are cared for in the public health sector, 33% (120) are treated in pediatric hospitals. Regarding public and private entities, 43.4% are attended in public institutions and 56.8% do so in private institutions.

### 3.2. Clinical data

The age at onset of symptoms in SMA 1 is 3 months (2.00–4.00 IQR), for SMA 2, 11.7 months (7.00–15.00), for SMA 3, 24 months (8.00–51.00) and for SMA 4, 18 years (18–19).

The age at diagnosis is expressed in months - years, median age and IQR. For SMA 1: 5.2 months (IQR 3.37–7.43), for SMA 2: 18.68 months (IQR: 14.18–27.77), for SMA 3: 49.87 months (IQR 29–118) and for SMA 4, 28 years (IQR 26–34).

Of the patients who died 34, 10% of the total were SMA 1. In SMA 2 and 3, one death was recorded in each group and none in SMA 4. Of all, 53% of the deaths occurred in the first year of life.

Regarding the copies of *SMN2*, only 111 (33.4%) of the patients had their quantification performed. In SMA 1, 19 of 20 have 2 copies of *SMN2*. In SMA 2, 16 of 52 patients reported 2 copies and 32 presented 3 copies. In SMA 3, 17 of 26 patients report 3 copies of *SMN2.*

Regarding the current motor capacity, 83 patients of SMA 1 did not have any motor milestones, only 14 patients achieved head support, one sitting and another walking. More than 50% of SMA 1 are tracheostomized (69 patients) and 6 patients on non-invasive ventilation. Regarding feeding, 45 patients have a nasogastric tube and 42 are gastrostomized.

If we take SMA type 2 into account, only 7 patients have not achieved any motor milestone, 46 achieve head support, 80 sit down and 16 walk. Only 2 patients are tracheostomized and 7 on non-invasive ventilation. Regarding feeding, 3 do so through a nasogastric tube.

In relation to SMA 3, more than 50% (53 patients) walk, 25 achieved sitting position and 3 patients only cephalic support. None of them require support for feeding or breathing.

**Figure 2:**
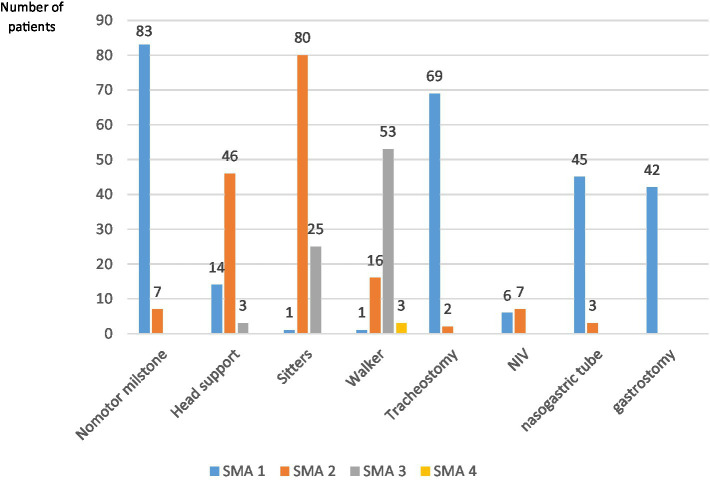
Current status of patients. The number of patients for each subtype of spinal atrophy who reach major motor milestones is described, as well as those who require respiratory or swallowing support. NIV, non invasive ventilation.

All SMA type 4 patients walk, without needing any other support (Figure 2).

### 3.3. Access to standards of care

Regarding all therapies access, without discriminating subtype of SMA:

83.7% have physical therapy, 39.54% occupational therapy, 19.87% hydrotherapy, 27.10% standing frame, 24.39% speech therapy and 29.21% have cough assist.

If we take into account each subtype of SMA, separately:

In SMA 1, 81.9% have regular physical therapy, 46.5% occupational therapy, 27.3% use cough assist, 17.2% use a standing frame, 5.1% perform hydrotherapy, 30.3% speech therapy.

In SMA 2, 89.3% have access to physical therapy, 45.6% to occupational therapy, 40.3% use a cough assist, 38.3% use a standing frame.

In SMA 3, 79% perform physical therapy, 29.6% hydrotherapy, 21% occupational therapy, 19.8% use standing frame and 14.8% cough assist (Table 2).

**Table 2:** Medical care and Access to standards of care.

Health care item	Categories	% Of patients
Medical coverage	Prepaid medicine	24.1%
	Social security	50.6%
	Public	22%
	Prepaid and social security	3%
Place of medical care	Pediatric Public Hospital	27.5%
	General Private Hospital	24.47%
	General Public Hospital	14.68%
	Private Office	14.68%
	Private Neurological Center	7.95%
	Private Pediatric Hospital	6.11%
	Private University Hospital	2.75%
	Public University Hospital	1.22%
	Rehabilitation Center	0.84%
Therapies access	Physical therapy	83.7%
	Occupational therapy	39.45%
	Hydrotherapy	19.87%
	Standing frame	27.10%
	Speech therapy	24.39%
	Cough assist	29.21%

Given the mixed health system in Argentina (prepaid medicine, social security and public), as well as the different profile of institutions (pediatric, general, university hospitals and others), the entire profile of coverage and places of care for patients is specified, as well as their access to therapies at the time of the study.

There is no data available on the relationship between access to therapies in relation to the corresponding medical indication, although it is probable that some require it in 100% of patients –physical therapy in SMA 1-and others may not be necessary in the majority of patients. Patients with a subtype of SMA – speech therapy in SMA 3.

### 3.4. Access to specific pharmacological treatment

Regarding the situation of specific treatment in Argentina, 199 patients are under treatment with nusinersen (47 SMA type 1, 104 SMA type 2 and 48 SMA type 3), 21 patients were infused with Onasemnogen, 13 were SMA type 1 and 8 SMA type 2. One child with SMA type 1 and 11 with SMA type 2 have been treated with Risdiplam (Table 3).

**Table 3:** Patients in specific pharmacological treatment.

	SMA type 1	SMA type 2	SMA type 3	SMA type 4
Nusinersen	47	104	48	0
Onasemnogene	13	8	0	0
Risdiplam	1	11	0	0
Total patients in specific treatment	59/99 (59.59%)	121/149 (81.20%)	48/81 (59.25%)	0%

The patients who have had access to treatment by SMA subtype are detailed, taking into account that 4 patients, 2 in SMA1 and 2 in SMA2, have accessed more than one treatment (Onasemnogene and nusinersen).

These results make a total of 228 patients with access to treatment (68%), taking into account that 4 patients received more than one treatment.

## 4. Discussion

The data obtained and reviewed allow us to have a specific overview of SMA in Argentina, as well as to detect items not included in it at the time of its creation and that need to be incorporated into the registry (scoliosis, scoliosis surgery, among others). We believe that the number of patients registered and reviewed is highly representative of the disease in Argentina, and it is likely that few patients are excluded from it. It is observed that a group of patients, mainly those with an older diagnosis, did not have a molecular study and had to be excluded.

Likewise, only 33.4% of the total have a count of *SMN2* copies and the data reported in this regard should be taken with caution since the majority of patients with SMA type 2 report having a considerable percentage of 2 copies of *SMN2* (30.7%), which is discordant for the habitual genotype–phenotype correlation of the disease described in the international literature and that studied by another Argentine group (13).

Within the total number of patients, 44.9% correspond to SMA type 2, this being the largest subgroup of patients. Without discriminating by type of SMA, 44% of the children are less than 10 years old, 27.7% of the patients are between 10 and 19 years old, and 28% are 20 or older. That is to say that about 1/3 of the patients are of adult age.

There is a gap between time of diagnosis and the onset of symptoms identifiable by the family. Added to this 25% of cases are diagnosed after 7, 27, and 117 months for SMA 1, 2, and 3, respectively. Despite the diagnostic delay being reported also in other series, it may be a critical point for the diagnosis and early access to treatment mainly in SMA 1 (14).

Most of the patients, 82.9%, are geographically located in 6 provinces. In them is 69.7% of the Argentine population according to INDEC (National Institute of Statistics and Censuses). It must be taken into account that most of the specialized centers are located in a limited number of cities, which may also influence this distribution in addition to the demographic factor.

The deceased patients correspond mainly to SMA 1 and especially in the first year (52%). It should be remembered that an important subgroup corresponds to the era prior to specific treatment in which a therapeutic option was only palliative care for patients without advancing in respiratory assistance and this reason may be related to the difference with other natural history series that report 40% deaths in the first year (15).

In the period 2014 to 2018, a media of 16.57 children diagnosed with SMA (range 9–22) were included annually. Taking into account the statistics of live newborns INDEC, an incidence between 1.2 and 3.1 per 10,000 live births is estimated. This data may be relevant in terms of a future newborn screening followed by treatment for SMA, since it has not yet been incorporated into the country’s routine newborn screening.

If the population projected by INDEC for 2021 is taken into account (based on projections, since the 2021 population census was suspended due to the COVID19 pandemic) and the 300 living patients present in the registry, we would find 6.5 patients with SMA included in the present protocol for every 1,000,000 inhabitants (16).

For pharmacological treatment 68% receive or received specific therapy, mainly SMA 2 patients and with nusinersen. There are factors that can condition: (1) Many patients with SMA 1 with many years of evolution and advanced stage that may have influenced the indication for treatment, unlike the group of SMA2 patients with a better general state at the time of treatment appearance, (2) Nusinersen has been used in the country since 2017 since our group began using it under the compassionate use regime and in 2020 it was the first to be approved by ANMAT, and (3) There are only partial coverage/reimbursement agreements from the Ministry of Health for SMA 1 and 2 for nusinersen and for SMA 1 under 9 months of age for Onasemnogene, which makes access to treatment difficult for patients with indications for the treatment, mainly in SMA type 3.

In a second stage, our group studies the impact of the different drugs on the clinical status of the patients, being in the final stage of data review.

The presence of a selection bias related to the fact that patients are voluntarily included in the registry should be considered. We also highlight information bias related to two aspects: (1) data reported by patients and their families and not derived from a structured medical report and (2) in older patients, there is a long time between diagnosis and inclusion in the registry, which can generate imprecise information.

## 5. Conclusion

The data from this study show a very broad profile of the SMA situation in the country, providing demographic, clinical, and access to treatment data. From it emerge data such as the total number of cases by SMA subtype, geographic distribution, ages of prevalence, type of access to medical coverage and places of care, which can be useful for planning medical care and the resources necessary for this. Disease.

At the same time, it will allow making projections regarding the possibility of implementing research protocols for new drugs, as well as starting the path towards the future implementation of neonatal screening.

## Author contributions

All authors listed have made a substantial, direct, and intellectual contribution to the work and approved it for publication.

## Funding

This research was supported by Novartis Argentina. Novartis also provided expert input in the development of the study design. The funder of the study had no role in data collection, data analysis, data interpretation, or writing of the report.

## Conflict of interest

The authors declare that the research was conducted in the absence of any commercial or financial relationships that could be construed as a potential conflict of interest.

## Publisher’s note

All claims expressed in this article are solely those of the authors and do not necessarily represent those of their affiliated organizations, or those of the publisher, the editors and the reviewers. Any product that may be evaluated in this article, or claim that may be made by its manufacturer, is not guaranteed or endorsed by the publisher.
